# Towards improving the accuracy of aortic transvalvular pressure gradients: rethinking Bernoulli

**DOI:** 10.1007/s11517-020-02186-w

**Published:** 2020-05-26

**Authors:** Benedikt Franke, J. Weese, I. Waechter-Stehle, J. Brüning, T. Kuehne, L. Goubergrits

**Affiliations:** 1 Institute for Imaging Science and Computational Modelling in Cardiovascular Medicine, Charité Universitaetsmedizin Berlin, Augustenburger Platz 1, 13353 Berlin, Germany; 2 Philips Research Laboratories, Hamburg, Germany; 3 DZHK (German Centre for Cardiovascular Research), Partner Site Berlin, Berlin, Germany; 4 Einstein Center Digital Future, Berlin, Germany

**Keywords:** Aortic valve stenosis, Bernoulli equation, Computational fluid dynamics, Aortic valve area, Transvalvular pressure gradient

## Abstract

The transvalvular pressure gradient (TPG) is commonly estimated using the Bernoulli equation. However, the method is known to be inaccurate. Therefore, an adjusted Bernoulli model for accurate TPG assessment was developed and evaluated. Numerical simulations were used to calculate TPG_CFD_ in patient-specific geometries of aortic stenosis as ground truth. Geometries, aortic valve areas (AVA), and flow rates were derived from computed tomography scans. Simulations were divided in a training data set (135 cases) and a test data set (36 cases). The training data was used to fit an adjusted Bernoulli model as a function of AVA and flow rate. The model-predicted TPG_Model_ was evaluated using the test data set and also compared against the common Bernoulli equation (TPG_B_). TPG_B_ and TPG_Model_ both correlated well with TPG_CFD_ (*r* > 0.94), but significantly overestimated it. The average difference between TPG_Model_ and TPG_CFD_ was much lower: 3.3 mmHg vs. 17.3 mmHg between TPG_B_ and TPG_CFD_. Also, the standard error of estimate was lower for the adjusted model: SEE_Model_ = 5.3 mmHg vs. SEE_B_ = 22.3 mmHg. The adjusted model’s performance was more accurate than that of the conventional Bernoulli equation. The model might help to improve non-invasive assessment of TPG.

**Graphical abstract:**
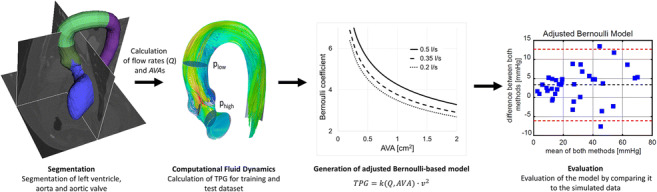
Processing pipeline for the definition of an adjusted Bernoulli model for the assessment of transvalvular pressure gradient. Using CT image data, the patient specific geometry of the stenosed AVs were reconstructed. Using this segmentation, the AVA as well as the volume flow rate was calculated and used for model definition. This novel model was compared against classical approaches on a test data set, which was not used for the model definition.

Processing pipeline for the definition of an adjusted Bernoulli model for the assessment of transvalvular pressure gradient. Using CT image data, the patient specific geometry of the stenosed AVs were reconstructed. Using this segmentation, the AVA as well as the volume flow rate was calculated and used for model definition. This novel model was compared against classical approaches on a test data set, which was not used for the model definition.

## Introduction

The prevalence of valvular heart diseases (VHD) is approximately 2.5% (95% confidence interval 2.2–2.7%) [23]. This prevalence significantly increases with age. While the prevalence in 18–44-year olds is only 0.7%, it is 13.3% and thus more than ten-fold larger for people above the age of 75 years [27]. In industrial countries, the majority of valvular diseases are degenerative, whereas rheumatic diseases, which are still a major burden in developing countries, have fallen dramatically [23, 31]. Results of the EuroHeart Survey [17] suggest that a substantial burden of valvular diseases, at least in Europe, exists. As degenerative valve diseases become more prevalent in older age, the overall increasing life expectancy results in large numbers of affected patients.

Among VHD, aortic valve stenosis is the most common disease in developed countries. According to the VHD Guideline, classification of disease severity and respective medical therapy recommendations for aortic valve stenosis should be based on multiple criteria [22]. These include findings of physical examinations and data from comprehensive transthoracic echocardiography. Cardiac catheterization is recommended only in symptomatic patients with inconclusive non-invasive test results [22]. Three parameters are used to quantify the severity of valvular aortic stenosis: maximal velocity *v*_max_, mean pressure drop Δ*p*_mean_, and aortic valve area (AVA). All these parameters can be estimated based on the assessment of blood velocities in the vicinity of the aortic valve (AV) using Doppler echocardiography. Note, however, that different AVA definitions (geometric or flow based) with different cut-offs for decision making exist according to clinical guidelines [3]. The transvalvular pressure drop is commonly referred to as transvalvular pressure gradient (TPG). While velocities can directly be measured using echocardiography, pressures are estimated using the Bernoulli equation, which describes the relationship between TPG and the maximal velocity in the valve region *v*_max_ as TPG = *k* × (*v*_max_)2, where *k* = 4. Here, the equation for dynamic pressure (0.5 × ρ × *v*_max_2) is simplified to 4 × *v*_max_2 due to the density of blood (*ρ* ~ 1050 kg/m^3^) and a conversion from Pascal to mmHg (1 mmHg = 133.32 Pa) resulting in a dimensional coefficient *k* being approximated to 4 in the clinically used Bernoulli equation. Two assumptions are made by using the Bernoulli equation. First, the temporal acceleration and deceleration of the blood flow are negligible (the flow is steady). Second, only the flow along one streamline with maximal velocity passing the aortic valve is considered. It is also important to note that according to clinical guidelines, peak-systolic TPG_ps_ and cycle-averaged TPG_mean_ are considered to be equivalent measures. The relationship between the mean and the maximum pressure drop is described as TPGps = *a* × *TPG*_mean_, with the coefficient *a* lying in the range of 1.56 and 1.68 [22]. AVA is an additional parameter involved in the assessment of the severity of the aortic valve stenosis, which can be estimated using various approaches including echocardiography [27, 30]. Even though AVA is also a recommended parameter in clinical guidelines, this parameter cannot be used equivalently to TPG and the maximal velocity to assess the severity of a stenosis. This becomes evident, as significant differences in event-free survival rate were found for different aortic *v*_max_ ranges, whereas no significant difference was found for patients with different AVAs [29].

Thus, an accurate assessment of TPG is essential for the prognosis of the VHD progress and the medical treatment decision. Current methods of non-invasive assessment of TPG are mainly based on the Bernoulli equation. However, efforts were made to improve the accuracy of the Bernoulli-based approach by introducing a coefficient depending on the stenosis degree [26]. Also, alternative methods based on magnetic resonance imaging or computational fluid dynamics (CFD) were proposed [8, 15].

While the Bernoulli equation represents a gross simplification of the complex relationship between static pressure and flow velocity, studies reported good correlations between Bernoulli-based and catheter-measured TPG, the latter being considered as gold standard for TPG assessment [13]. The Bernoulli approach is considered to be equivalent to catheterization in clinical practice. Although significant linear correlations between Bernoulli-based and catheter-based TPG measurements were found in various studies, the general accuracy of the Bernoulli-based approach is questioned [21, 26]. Several studies reported that the Bernoulli equation is either overestimating or underestimating invasive measurements suggesting that it is insufficient to use a constant coefficient *k* in the Bernoulli equation. The Bernoulli equation, which was derived from the principle of conservation energy, is valid only for steady flows without viscous losses in one dimension. Therefore, neither energy losses from friction and dissipation as well as turbulence and effects of complex flows with strong secondary flow features nor the transient effects of acceleration and deceleration of the blood flow are taken into account [7, 34].

The inaccuracy of the Bernoulli approach is well exemplified by a study of Danielsen et al. [6]. They compared Doppler- and catheter-measured TPG ranged from 16 to 144 mmHg in 54 patients with aortic valve stenosis. Doppler echocardiography significantly overestimated the catheter-based TPG by 5.0 mmHg with a standard deviation of the differences of 12.0 mmHg. In 5 of 54 cases, the bias was larger than 25.0 mmHg. Similar inaccuracy was found in another study, which found a mean difference of 5.0 mmHg and a standard deviation of the differences of 13.0 mmHg [14].

In the era of precise medicine, diagnostic procedures need to be as precise as possible. It is therefore important to find improvements or alternatives to counteract the inaccuracy of the Bernoulli equation. The aim of this study was to evaluate a novel, non-invasive model to precisely assess TPG based on AVA and the volume flow rate. The novel model is aimed to allow a more robust and precise estimation of transvalvular TPG compared to the current clinical approach using the Bernoulli equation. Here, the basic idea was (1) to fit the coefficient *k* of the Bernoulli equation as a function of the volume flow rate (Q) and AVA instead of using the constant coefficient equalling 4 as currently done in the clinical practice and (2) to use AVA averaged velocity instead of the AV maximal velocity. The study was based on spatially well-resolved CFD simulations of patient-specific AV hemodynamics simultaneously providing both velocity and pressure fields. Velocity fields are used for TPG estimation, whereas pressure fields provide true TPG for error assessment.

## Materials and methods

### Data description and geometry pre-processing

This retrospective study is based on a dataset of 21 patients featuring mild and moderate aortic valve stenoses which was published earlier [32]. Additionally, the image data of four other patients were used to generate a test dataset, which will be described later in this section. The geometric models were based on data from computed tomography angiography (CTA) scans. Briefly, CTA data with an in-plane resolution between 0.31 × 0.31 and 0.89 × 0.89 mm^2^ and a slice thickness between 0.34 and 0.75 mm and a temporal resolution of 10 equidistantly phases per heart cycle were acquired. Using this data, the patient-specific anatomy was segmented for all 10 acquired phases. These segmentations were done semi-automatically using a shape-constrained deformable model that was detailedly described earlier [32]. First, the anatomical structures of interest were detected using a generalized Hough transformation. Afterwards, separate parametric models were adapted to the image data. For this, two parametric models were combined: a parametric model of the whole heart described by Ecabert et al. [9] as well as a parametric model of the tricuspid AV described by Weese et al. [32]. Each leaflet of the parametric AV model is represented by a double-layered “wedge” with corresponding vertices between both layers in order to prevent intersections between those layers. This geometric representation of AV leaflets facilitates the mesh generation for CFD flow modeling.

Furthermore, projected AVA and left ventricular (LV) volumes were provided for each phase. In this study, not only the heart phase of maximal flow and maximal AVA, but all systolic heart phases during which the AV was open were investigated using quasi-steady flow simulations. This approach was chosen to increase the number of different geometries that can be used for the subsequent definition of the model. The number of phases with open AV varied between 1 and 4. Finally, the dataset contained 59 geometries.

We excluded one patient from the study due to problems regarding the surface mesh of the parametric model. This model could not be processed in the subsequently described steps of numerical mesh generation.

### Numerical simulation of the transvalvular pressure gradient

In this study, the patient-specific hemodynamics were numerically calculated using a finite volume discretization. For this purpose, a numerical mesh representing the volume of interest had to be generated. The geometries provided by Weese et al. [32] were processed with Meshmixer (v. 11.0.544, Autodesk, California, USA). First, the initial surface mesh was smoothed and checked for possible intersections, holes, and manifolds. Next, the LV, the aorta, and the AV were extracted from the entirety of the cardiovascular model (right heart, pulmonary artery, left atrium with veins and mitral valve). The LV was cut manually about 20–30 mm below the AV, creating the left ventricular outflow tract (LVOT) inlet plane. For this, the LVOT inlet plane was set aiming to be parallel to AVA as well as orthogonal to the LVOT’s centerline.

**Fig. 1:**
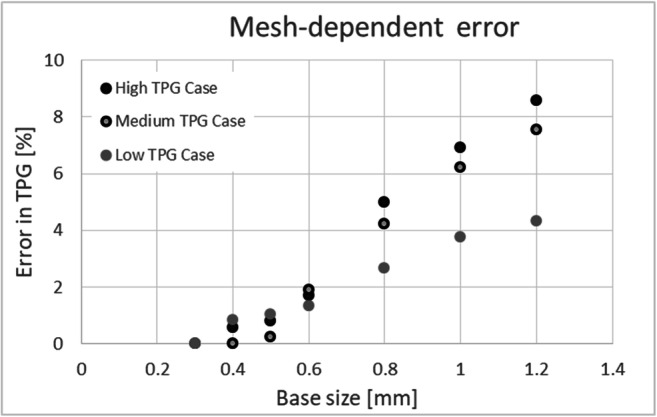
Mesh-dependent error in three representative cases with low, medium, and high TPG

**Fig. 2:**
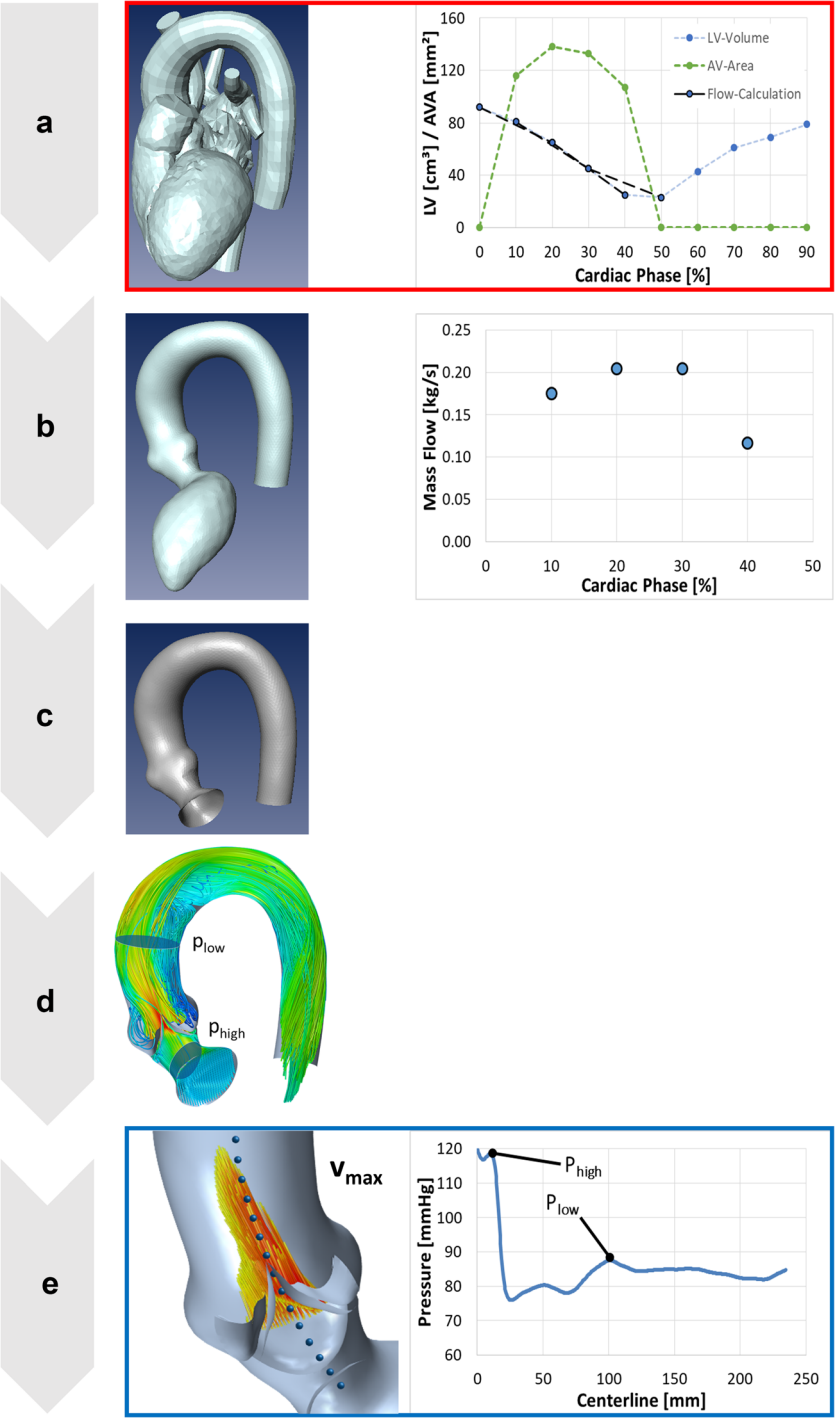
Processing pipeline: segmented cardiovascular geometries and the information of AVA and the LV volumes were required as input (**a**). The domain of interest was extracted from the heart model (**b**, left). The LV was cut and the remaining domain was meshed (**c**). Flow rates were calculated from the LV volume data (**b**, right). Quasi-steady simulations were performed (**d**) and post-processed (**e**)

Polyhedral volume meshes were created using the integrated meshing algorithm of STAR-CCM+ (v. 13.02.011, Siemens PLM Software Inc., Texas, USA). To identify the required mesh resolution to achieve mesh-independent results for TPG, a mesh-independency study with different cell sizes (seven mesh resolutions per case) in a range from 0.3 to 1.2 mm was performed for three cases (low TPG of 7 mmHg, middle TPG of 38 mmHg, and high TPG of 78 mmHg) representing the expected range of TPG (Fig. 1). For all cases, a base size of 0.8 mm was enough to achieve a resolution-based error of TPG less than 5% compared to the value calculated with the finest mesh. The error decreased below 2% for a base size of 0.6 mm. For most cases, we used a finer mesh than required by the defined criterion resulting in a mesh of 0.8 to 12 million cells. This is due to the fact that the finer mesh was necessary to meet the criteria set for convergence of numerical residuals for momentum and continuity, as described at the end of this section. A boundary layer mesh was generated using 5 prism cell layers in order to correctly resolve the near-wall region without severely increasing the overall mesh size. Each layer’s height was 120% of the previous layer’s height. The overall thickness of the boundary layer was 33% of the base size. Near the AV, a local mesh refinement was defined, so that the base size in this region was 50% of the global base size. The minimal cross-section of the aortic stenosis was resolved using 3000 to 4000 elements. At the LVOT inlet boundary, a case-specific mass flow rate was applied. This was calculated based on the available information of the LV volume for each phase. LV volume change between phases was calculated and divided by the time step between two subsequent phases (Fig. 2). Since no patient-specific heart rates were available, a time step of 95 ms, corresponding to a heart rate of 63 bpm, was used for all cases in order to calculate flow rate from the measured left ventricle volumes. Therefore, the average and standard deviation of simulated flow rates of the 59 cases (approximately 3 phases of the systole per case) was 0.217 ± 0.109 l/s.

**Fig. 3:**
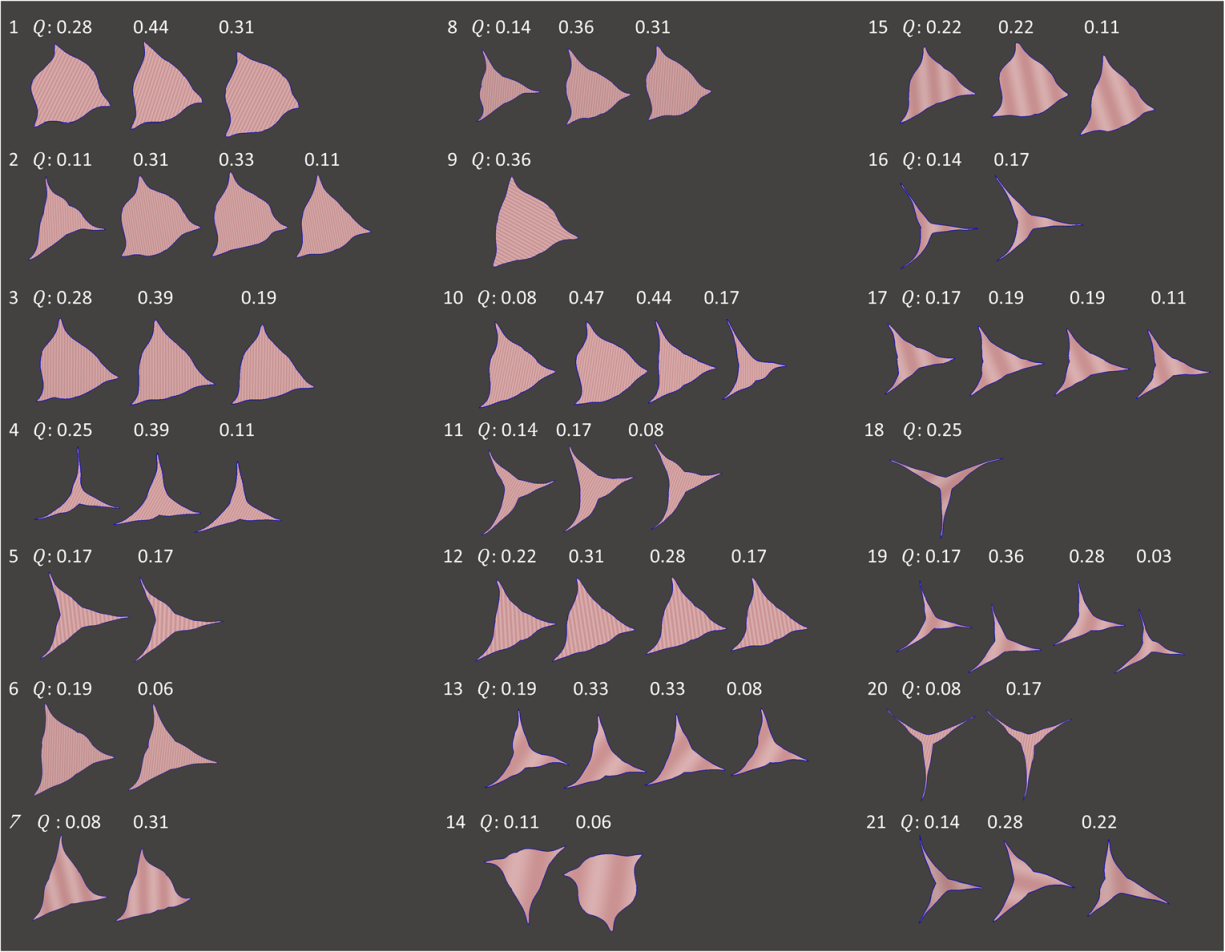
Shapes of projected AVAs for 59 simulated geometries obtained from 21 patients. The unaltered volume flow rates Q that were estimated using the left ventricle volumes are specified above each geometry in liters per second

During the previous published numerical investigation of the dataset used in this study [32], only 21 peak-systolic pressure drops were calculated. Calculated TPG values were below 30 mmHg and only three values were above 20 mmHg. Thus, only small and mild TPG were expected in our study as well. In order to include higher TPG values for the subsequent model specification, reconstructed cases were also simulated with flow rates increased by factor 1.5, 2.5, and 3.0. The aim was to increase the number of cases with a pressure drop above 30 mmHg but avoid non-physiologic high flow rates. Using this approach, artificial peak-systolic volume flow rates were generated, allowing to better sample the expected range of physiologically occurring flow rates. One case was excluded from the study. Here, an unrealistic high velocity and pressure drop was observed (*v*_max_ > 8.0 m/s and TPG > 200 mmHg) probably caused by errors in the volumetric analysis. In total, 135 simulations using 59 different geometries of the AV in 21 aortic geometries were performed (see Fig. 3). The average and standard deviation of simulated flow rates for these 135 cases was 0.318 ± 0.216 l/s.

Vessel walls were defined as rigid and a no-slip condition was applied. The end of the descending aorta was truncated perpendicular to the centerline. Here, a pressure outlet was specified with a constant pressure of 0 mmHg.

Numerical simulations under quasi-steady conditions were performed using the commercial software STAR-CCM+. A finite volume formulation was used for discretization of the Navier-Stokes equations describing the continuity and momentum of incompressible fluids. An implicit steady-state solver was used, and the density of the blood was set to ρ = 1050 kg/m^3^ while considered incompressible. Dynamic viscosity was modeled by the generalized Carreau-Yasuda model [18]. The threshold kinematic viscosity of the viscosity model was η = 0.0035 Pa ∗ s. The model was used with parameters proposed by Abraham et al. [1]. Reynold numbers (*Re*) were calculated using η, the characteristic size of the stenosed aortic valve defined as characteristic diameter $\mathrm{D}=2{\left(\frac{\mathrm{AVA}}{\pi}\right)}^{0.5}$ and the average velocity (u) in this minimal cross-section ($\operatorname{Re}=\frac{\rho \times \mathrm{u}\times \mathrm{D}}{\eta }$). Resulting *Re* numbers ranged from 500 to 14,000, with an average of 3944. Although *Re* is quite low in some cases, it has been shown that instabilities in jet flows still occur at such low *Re* values, and it is therefore necessary to use a turbulence model [34]. Additionally, the influence of a turbulence model on the TPG in cases with low *Re* was investigated. Six cases with *Re* between 500 and 2000 were simulated using a laminar solver. In all six cases, the differences between laminar and turbulent solvers for TPG were below 0.3 mmHg. Therefore, we decided to calculate all results for simulation using a turbulence model. Turbulence was modeled using a realizable *k*-epsilon two-equation turbulence model. As convergence criterion for inner iterations, a value below 10^−3^ for the residuals was set for the conservation of mass and the conversation of linear momentum and a threshold of 10^−2^ was specified for the residuals of the turbulent dissipation rate.

For each simulated geometry, a centerline was created using ZIBAmira (v. 2015.28, Zuse Institute Berlin, Berlin, Germany). Along this centerline the cross-section averaged static pressure was computed. Data analysis was done with MATLAB (v. 2016a, Mathworks, Inc., Natick, USA).

To compute the exact TPG, the pressure upstream of the stenosed valve (*p*_high_) was measured within the smallest diameter in the region between the LVOT and the AV. The pressure value was averaged across the respective cross-section. The static pressure downstream the stenosis (*p*_low_) was measured by averaging over cross-sections at the centerline position in the ascending aorta where the pressure recovery due to deceleration of the blood was maximal. This position usually lied approximately 40 to 60 mm behind the AV. This exact, CFD-based TPG was calculated as TPG_CFD_ = *p*_high_ − *p*_low_ (Fig. 2e).

The maximal velocity *v*_max_ in the valve region was derived from the numerical simulations by calculating the maximal velocity component in the direction perpendicular to the AV’s annulus plane. This approach was chosen to allow high comparability to transthoracic echocardiography, where only this normal component of the velocity is measured. Using this velocity value, the Bernoulli-based TPG_B_ was calculated as TPG_B_ = 4 × *v*_max_^2^. This definition of TPG neglects pressure recovery, while pressure recovery was considered for the definition of TPG_CFD_, which is considered to be the ground truth. According to Garcia and Kadem [30], TPG assessed by Bernoulli is a so called TPG_max_, whereas TPG considering the pressure recovery, as was calculated by CFD, is TPG_net_. The pressure recovery neglection is a common clinical practice since the assessment of the pressure recovery by echocardiography is challenging. The impact of this design decision is evaluated in the discussion chapter.

There are three different methods of the AVA measurement according to [10]: First, by measuring planimetric AVA at a defined position of the valve. Second, by using the effective orifice area (EOA), which is the area defined by the position of the vena contracta, the region of the smallest diameter of the blood flow. Third, by projecting of either the whole AV or the leaflet rims. In this work, we projected the leaflet rims and thus measured AVA. The resulting AVAs ranged from 0.64 to 4.37 cm^2^ with an average and standard deviation of 1.87 ± 0.87 cm^2^.

### Adjusted Bernoulli model for prediction of the transvalvular pressure gradient

Currently, the Bernoulli-based approach for calculation of the pressure gradient across an aortic stenosis uses an equation with a constant coefficient and the square of the maximal velocity magnitude: TPG_B_ = 4 × *v*_max_^2^. Here, the constant coefficient of 4 is an approximation resulting from the density of blood and a conversion from Pascal to mmHg. However, Oshinski et al. found that this constant coefficient has to be adjusted to the given disease as well as to the disease severity [26]. They proposed adjusted coefficients based on the degree of stenosis of an aortic coarctation. While they reported a monotone increase in the coefficient when the degree of stenosis increased, the reported coefficients varied from 2.3 to 4.9.

Following this approach, we hypothesize that an adjusted coefficient might also allow a more accurate prediction of the pressure gradient across an aortic stenosis.

We propose a power law model to describe the relationship between the coefficient *k* used in the Bernoulli equation as a function of AVA and volume flow rate Q:

1 $$ k=c\times {\mathrm{AVA}}^{\upalpha}\times {Q}^{\upbeta} $$

Clinically, the Bernoulli equation is applied to the maximal velocity, usually measured using echocardiography. We, however, propose using the average velocity, as the average velocity already is defined as the ratio of volume flow rate and cross-sectional area. Therefore, no additional measurement for the maximal velocity is required. The equation for Bernoulli-based estimation of TPG using our proposed approach would be

2 $$ {\mathrm{TPG}}_{\mathrm{Model}}=c\times {\mathrm{AVA}}^{\upalpha}\times {Q}^{\upbeta}\times {\tilde{v}}^2=c\times {\mathrm{AVA}}^{\upalpha}\times {Q}^{\upbeta}\times {\frac{Q}{\mathrm{AVA}}}^2=c\times {\mathrm{AVA}}^{\upalpha -2}\times {Q}^{\upbeta +2} $$

First, a power law fit using *α′* = *α* − 2 and *β′* = *β* + 2 was calculated using the curve fitting toolbox provided by MATLAB, and then, *α* and *β* were calculated, respectively. Here, the coefficients and exponents c, α′, and β′ were to be estimated using the nonlinear least-square method without a robust function combined with the trust-region algorithm. As we aimed to develop a method assessing accurately ground truth TPG, TPG_Model_ is, similar to TPG_CFD_, a pressure gradient value taking into account pressure recovery. Hence, this is a TPG_net_ value.

### Test data for proof-of-concept of the model for prediction of the transvalvular pressure gradient

**Fig. 4:**
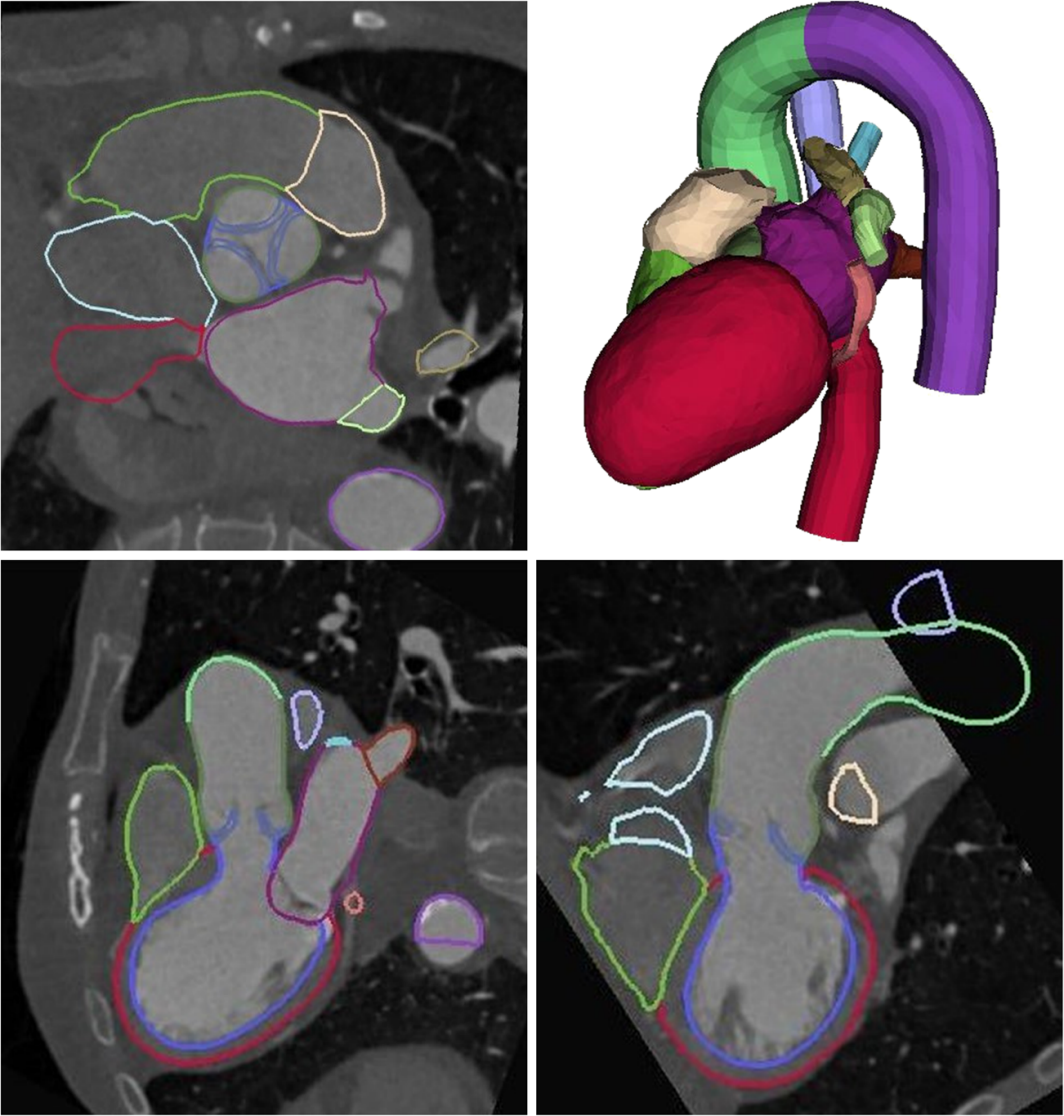
Exemplary visualization of three orthogonal cross sections of CT images featuring an open AV. The colored contours represent the segmentation of the different compartments of the heart. In the upper right panel, the 3D geometry of the segmented compartments is shown

Using the same procedure as described above, CT images of four additional patients who also suffered from aortic stenosis were processed to generate 3D models of the LVOT, the aorta and the AV. For each patient, four states featuring an open AV were reconstructed, resulting in 16 geometries. The original as well as two elevated flow rates were simulated per geometry, resulting in 36 simulations. The CT images as well as the segmentations generated are visualized in Fig. 4.

These 36 simulations were used as a test dataset on which the proposed model for estimation of the pressure gradient across the AV was evaluated and compared against the Bernoulli-based estimation of the pressure gradient. Therefore, velocity and pressure field were calculated using the numerical method as described above. Then, the exact TPG_CFD_ was calculated using the numerical simulations. Additionally, TPG was predicted using the model which was parameterized using the training data (TPG_Model_) as well as the Bernoulli equation (TPG_B_). To compare these predictions against each other, the SEE was calculated for each estimate as well as the number of patients which were misclassified regarding the clinical threshold of 40 mmHg. Here, *n*_FP_ is the number of false positive classifications, where TPG_CFD_ was below the threshold but the model predicted a TPG above the threshold and *n*_FN_ is the number of false negative classifications, where the model failed to correctly predict a TPG above the threshold.

This study was carried out according to the principles of the Declaration of Helsinki and approved by the local ethics committee (Ethics committee: Charité-Universitätsmedizin Berlin). Written informed consent was obtained from the participants.

## Results

### Results for the training data set

**Fig. 5:**
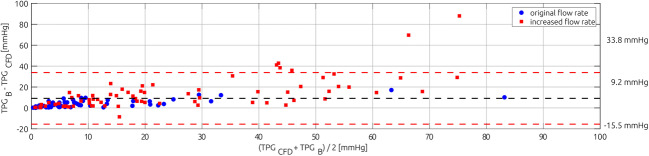
Bland-Altman plot comparing Bernoulli-based TPG_B_ to CFD-based TPG_CFD_ for all 135 cases. Fifty-nine cases with unaltered flow rates are marked blue

Figure 5 shows a Bland Altman plot comparing Bernoulli-based TPG_B_ and CFD-based TPG_CFD_ for all 135 simulated cases. Here, the 59 simulated cases with unaltered flow rates derived from segmented LV volume measurements are highlighted blue, while all simulations using increased volume flow rates are highlighted red. The averaged difference in TPG between both methods was 9.2 mmHg with a standard deviation of 12.6 mmHg. Averaged TPG_CFD_ of the investigated cases was 14.1 mmHg and had a standard deviation of 15.8 mmHg. Only 8 of 59 TPG_CFD_ values calculated using the unaltered flow rates were above 20.0 mmHg. Therefore, additional simulations using increased volume flow rates, as described earlier, were conducted. Considering all 135 simulations, 38 cases had a TPG_CFD_ above 20.0 mmHg. The linear correlation between TPG_B_ and TPG_CFD_ had a coefficient of determination of *R*^2^ = 0.78 and a standard error of estimate of SEE = ± 10.6 mmHg.

### Novel adjusted Bernoulli model

The information of all 135 simulations included in the training data set was used to determine coefficients of the proposed model. The resulting equation was

3 $$ {\mathrm{TPG}}_{\mathrm{Model}}=3.007\times {\mathrm{AVA}}^{-0.373}\times {Q}^{-0.216}\times {\tilde{v}}^2 $$

**Fig. 6:**
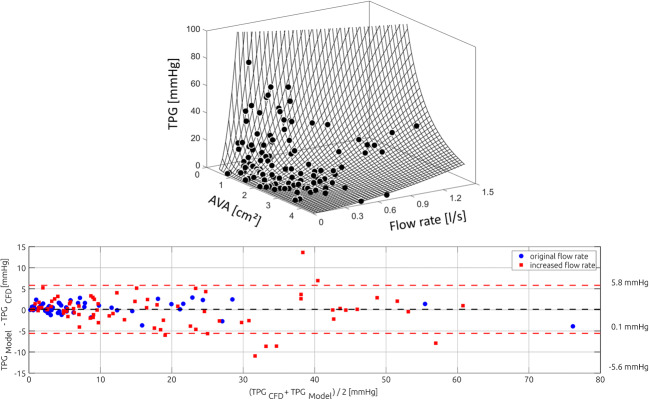
Scatter plot showing the training data used for sampling and the adjusted Bernoulli model that was fitted using this data (upper). In the bottom, the corresponding Bland-Altman plot comparing the adjusted Bernoulli model’s TPG_Model_ and the CFD-based TPG_CFD_ is shown

Here, the units of AVA and Q are cm^2^ and l/s, respectively. Confidence intervals of the three calculated coefficients of the eq. 3 and exponents were *c* = [2.696, 3.319]; *α* = [− 0.477, − 0.269], and *β* = [− 0.307, − 0.125]. The coefficient of determination was *R*^2^ = 0.966 and the standard error of estimate was SEE = ± 2.4 mmHg. The fit is shown in the top of Fig. 6. The mean difference between TPG_Model_ and TPG_CFD_ was 0.13 mmHg with a standard deviation of 2.91 mmHg (see Fig. 6, bottom).

### Performance of the novel model using the test data set

The mean and standard deviation of the exact TPG_CFD_ calculated using CFD for 36 test cases was 26.9 ± 18.3 mmHg. The mean and standard deviation of TPG estimations using either the model which was described previously (TPG_Model_) or the Bernoulli equation (TPG_B_) were 30.2 ± 19.2 mmHg and 44.2 ± 30.4 mmHg.

**Fig. 7:**
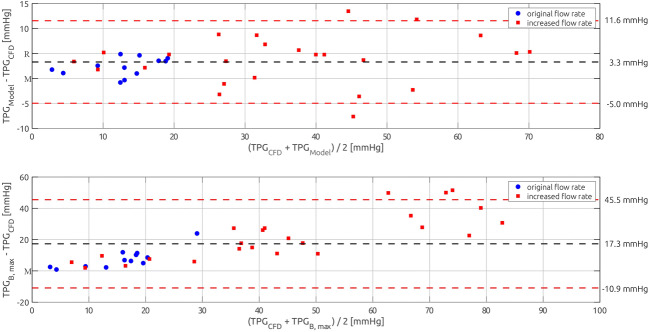
Bland-Altman plots comparing the numerically calculated pressure drop (TPG_CFD_) against the adjusted Bernoulli model (TPG_Model_, upper panel) as well as the Bernoulli-based estimation of the pressure gradient (TPG_B_, lower panel)

Both predictive models overestimated the exact TPG_CFD_. According to a Shapiro-Wilk test, the differences between the predicted TPG_Model_ and TPG_CFD_ were normally distributed (*p* = .349), while the differences between TPG_B, max_ and TPG_CFD_ were not normally distributed (*p* < .001). Due to these inconclusive findings, a non-parametric Wilcoxon signed-rank test was used to investigate, whether differences between the exact TPG_CFD_ and the model estimates were significant. Both models significantly overpredicted TPG_CFD_ with *p* < .001. Here, the mean difference between TPG_Model_ and TPG_CFD_ was 3.3 mmHg, while the mean difference between TPG_B_ and TPG_CFD_ was 17.3 mmHg (see Fig. 7).

The model estimates were compared against the exact TPG_CFD_ with respect to the correlation (*r*, *R*^2^) between exact and estimated TPG, the standard error of estimate (SEE) as well as the false positive and false negative classifications by the estimations (*n*_FP_, *n*_FN)_. The proposed model showed an overall better performance than the Bernoulli-based estimation of TPG. While both estimates correlated well with the exact TPG_CFD_, the coefficient of correlation with TPG_Model_ was *r* = 0.98 (*R*^2^ = 0.95) and *r* = 0.95 (*R*^2^ = 0.89) for TPG_B_; there were large differences in the SEE. The proposed model featured a SEE of 5.3 mmHg, whereas the SEE of the Bernoulli-estimate using the maximal velocity was 22.3 mmHg, respectively. As all models significantly overpredicted TPG, no false negative classification, where the model predicted that TPG was below the treatment threshold while it was actually above, was observed. Four false positive classifications (11%) were observed by the proposed model, while the Bernoulli estimates resulted in 11 (31%) false positive classifications.

## Discussion

Expectedly, a significant correlation between the Bernoulli-based estimates of TPG (TPG_B_) and exact TPG, which was derived from the numerically calculated pressure fields (TPG_CFD_) was found for the test data set. TPG_B_ significantly overestimated the exact TPG_CFD_, with an average difference of 17.3 mmHg. The SEE between both methods was ± 22.4 mmHg. Considering that the clinically relevant TPG threshold, upon which a treatment is recommended, is 40 mmHg, this SEE results in a high relative inaccuracy of more than 50%. If the treatment decision is based on the maximal velocity (*v*_max_) and not TPG, the clinical threshold is 4 m/s equalling a TPG_B, max_ of 64 mmHg. This still relates to an error of approximately ± 35%.

The linear correlation between Bernoulli-based and catheter-based measurements of TPG was demonstrated several times [4, 13, 19]. In these studies, the coefficients of correlation lay in a range between 0.78 and 0.98, which correlates well with our results (*r* = 0.95). In contrast, large variability of findings regarding differences between Bernoulli- and catheter-based TPG varying between 4.6 mmHg underestimation and 22 mmHg overestimation was published [4, 13].

Also, high inaccuracies of Bernoulli-based TPG estimation, which were in the same order as was found in our study, were reported: Lima et al. [18] reported an accuracy of Bernoulli-based TPG compared to catheter measurements with a SEE = ± 7.0 mmHg. However, Rijsterborgh et al. reported that the differences between both methods could be as large as 25 mmHg [28], whereas Goli et al. reported a SEE = ± 10.0 mmHg [11]. The proposed adjusted Bernoulli model for estimation of TPG shows improved performance compared to Bernoulli-based estimation of TPG. The calculated coefficient of determination of the fit was *R*^2^ = 0.95, while the coefficient of determination of the Bernoulli-based TPG was *R*^2^ = 0.89. Additionally, the accuracy of the adjusted Bernoulli model was higher than that of the Bernoulli-based prediction: SEE_Model_ = ± 5.3 mmHg vs. SEE_B_ = ± 22.4 mmHg. The Bernoulli-based prediction resulted in 11 false positive classifications out of 36 test cases (31%), where a TPG above 40 mmHg was predicted, while exact TPG_CFD_ was below this threshold. In contrast, the adjusted Bernoulli model resulted in only 4 (11%) false positive classifications. However, the adjusted model still significantly overestimated the exact TPG_CFD_, although the average difference was only 3.3 mmHg and thus five times smaller than that of the classical Bernoulli estimation.

This improved performance in predicting TPG can be explained by two major differences of the proposed approach compared to the Bernoulli equation: First, instead of only using *v*_max_, the approach takes both AVA and volume flow rate into account. Second, the Bernoulli equation is using *v*_max_ while the adjusted Bernoulli model uses the average velocity within the AV.

While neither TPG_B_ nor TPG_Model_ allow a direct consideration of the pressure recovery downstream of the stenosed valve, the adjusted Bernoulli model used for estimation of TPG_Model_ was developed on TPG values, where this pressure recovery was accounted for. This might explain a small portion of the improved performance of the adjusted Bernoulli model. However, the pressure recovery in the training data set was on average 3.0 mmHg and had a standard deviation of 3.5 mmHg, which is much smaller than the accuracy of TPG_B_ approach.

From a technical viewpoint, a stenosed AV equals a combination of two parts causing the pressure drop: a contraction and a sudden expansion. In order to estimate energy loss in technical applications, the Borda-Carnot equation is often used:

$$ \Delta \mathrm{E}=\xi \times 0.5\times \rho \times {\left({v}_1-{v}_2\right)}^2 $$

**Fig. 8:**
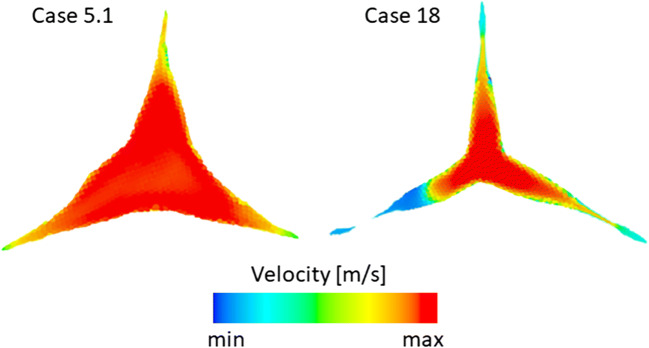
Color coded velocity magnitudes in the AVA plane showing a case with evenly distributed (left) as well as a case with a complex velocity profile. Case numbers refer to Fig. 3

This equation, which is derived from one-dimensional energy and momentum conservation equations, describes the mechanical energy losses of the fluid due to a flow expansion or contraction. The pressure drop is governed by the empirical loss coefficient (*ξ*), which ranges between 0 and 1. In case of a sudden expansion of a pipe, the loss coefficient is equal to 1 [2]. In other situations, the loss coefficient has to be determined by other means, usually empirically based on data obtained by experiments. This is in contrast with Bernoulli’s principle for non-viscous flows, where no irreversible losses exist and the total pressure is constant [2, 5]. Another difference of the Borda Carnot equation is that the pressure (energy) loss is proportional to the square of the mean velocity, not the maximal velocity as it clinically used by the Bernoulli approach. This difference is also exemplified using two cases from our study. In the first case (Fig. 8, left), the velocity magnitude is nearly evenly distributed within the AV’s cross section, almost equalling a constant velocity profile. For this case, the maximal velocity in the shown cross-section is only 7% higher than the mean velocity. Another case (Fig. 8, right) exhibits a velocity profile, where the maximal velocity is 39% higher than the mean velocity. Assuming these two cases have the same AVA and flow rate, same TPG for both cases is expected according to laws of fluid mechanics. However, TPG estimated for case 18 (Fig. 8, right) using the Bernoulli equation will be 69% higher, than in case 5.1 (Fig. 8, left). The model proposed by us mainly follows the Borda-Carnot approach.

While the Bernoulli equation does not consider AVA, the Gorlin equation defines a relationship between flow rate calculated usually with cardiac output (CO), heart rate (HR), AVA, the systolic ejection period (SEP), and mean TPG:

4 $$ \mathrm{AVA}=\frac{\mathrm{CO}\ }{\left(\mathrm{HR}\times \mathrm{SEP}\times {\left({\mathrm{TPG}}_{\mathrm{mean}}\right)}^{0.5}\times 44.3\right)} $$

Mean TPG is usually derived using the Bernoulli equation. The Gorlin equation seems to be similar to the adjusted Bernoulli model. However, the Gorlin equation proposes a linear relationship between AVA, Q, and *v*_max_, whereas the adjusted Bernoulli model found a non-linear relationship. Furthermore, the above-mentioned problem of using the maximal instead of the mean velocity is unsolved by the Gorlin equation. Altogether, these differences explain the inaccuracy of the Bernoulli-based approach.

Note that the Bernoulli equation estimates pressure drop for a streamline, whereas the pressure drop across a stenosed valve should be calculated by averaging over a set of streamlines. Thus, we are expecting that the Bernoulli equation using the valve area average velocity is better suited for assessment of TPG. Therefore, TPG_B, avg_ was calculated as:

$$ {\mathrm{TPG}}_{\mathrm{B},\mathrm{avg}}=4\times {\tilde{v}}^2=4\times {\left(\frac{\mathrm{Q}}{\mathrm{AVA}}\right)}^2 $$

for the test data set and compared against the exact TPG_CFD_. While this estimate still significantly overpredicted the exact TPG (*p* < .001), the average difference between the predicted values and TPG_CFD_ was 9.0 mmHg and thus half as large as the mean difference using the maximal velocity. Using the average velocity also resulted in a reduced SEE of 12.1 mmHg and a reduced number of false positive classifications of 6 out of 36 cases (17%). Therefore, as argued above, using the average velocity for the Bernoulli-based estimation of TPG will already result in an increased accuracy. However, calculating the average velocity also requires knowledge of the volume flow rate Q and the cross-sectional area of aortic stenosis AVA; therefore, the even better performing proposed adjusted Bernoulli model can be used as well.

We also analyzed the relationship between averaged and maximal velocities within the training data set and found that the mean and standard deviation of the ratio *ṽ*/*v*_max_ was 0.85 ± 0.082, while the range of this ratio was from 0.59 to 0.96.

A simple model for prediction of TPG across the AV was proposed. Alternatively, highly accurate computational fluid dynamics (CFD)-based approaches solving Navier-Stokes equations to calculate steady-state or transient 3D valve hemodynamics can be used [7, 32]. This approach, however, currently requires time-consuming procedures of the patient-specific geometry reconstruction and flow simulations. Respectively, clinical translation of the CFD approach is challenging. The method presented here is a compromise between accuracy of the CFD approach and low complexity level of the Bernoulli approach, which is expected to be easily translated into the clinical workflow followed by the clinical validation study.

### Limitations

The current study is based on 25 patient-specific cases. While this is a relatively small number of cases, we were able to use different valve segmentations for different phases of the systolic period. Furthermore, different flow rates were modeled, resulting in 171 simulations that were used in this study. These 171 simulations cover the expected range of pathophysiological situations regarding AVA and volume flow rates. The used deformable parametric model for AV segmentations was developed for tricuspid AVs only. Although the model was adapted to the Hounsfield information in the CTA images, it was still constrained with respect to the overall shape thus resulting in almost symmetrical AV geometries (see Fig. 3). A novel deformable model is necessary to segment bicuspid AVs, which form a common subgroup of aortic valve stenosis. The available data set includes only cases with mild and moderate aortic valve stenosis resulting in moderate TPGs. In order to add cases with higher TPG, simulations with higher flow rates were performed. Using the non-linear regression model for TPG estimation, which was developed using an adult cohort, we were unable to achieve non-dimensionalization with regard to the proposed model parameters. Accordingly, the model is not suited for investigation of scaling problems and is insensitive to some important scaling parameters such as valve shape or valve size, which could affect the model accuracy. This means that using the model for TPG estimation in infants should be validated separately. However, children only seldom suffer from aortic stenosis. Furthermore, a future study including patients with severe stenosis (AVA < 1 cm^2^) as well as cases with bicuspid aortic valves, thus considering larger valve size range, as well as the valve shape’s impact, is planned.

The current study is based on a simplified CFD model using quasi-steady flow simulations of the peak-systolic state as well as based on Reynolds-averaged Navier-Stokes equations (RANS) with a realizable *k*-epsilon two-equation turbulence model. While this turbulence model is known to be less accurate for simulation of turbulence parameters, modeling of turbulence in hemodynamic flows is a research topic on its own. Studies using LES or even DNS for simulation of blood flows [20, 24, 33] promise more accurate blood flow modeling. While no turbulent parameters were evaluated in this study, the chosen turbulence model might have an impact on the calculated TPG. However, the necessity for the use of more accurate turbulence model, which is also associated with higher computational costs, should be evaluated with regard to the results of the planned clinical validation study of the current model. The quasi-steady assumption, however, was shown to be valid for calculation of the AV’s resistance during peak-systole [16]. Furthermore, the same model was used for simulation of hemodynamics in patients suffering from aortic coarctation, another stenotic disease that affects the aorta. Here, simulated flow fields correlated well with velocity fields measured by 4D velocity encoded MRI [25] and accurately calculated the pressure drop across the stenosis [12]. The accuracy of the pressure drop calculation was validated against catheter-measured pressure drops. There are strong similarities between the flow observed in a stenosed aorta and the flow across a stenosed AV which is the subject of the present study. Especially, all other assumptions that are made in the present study, e.g., assuming rigid walls, neglecting the Windkessel effect of the aorta, were also applied in the study regarding aortic coarctation. As a validation against catherization was possible in case of the aortic coarctation, the model might also be suitable for simulation of the pressure gradient across an aortic stenosis. One reason that led us to keep most assumptions was that modeling aspects as the pulsatility of blood flow or the elasticity of the aortic wall will require to model these aspects in a patient-specific way, which will introduce further uncertainties. The numerical model is chosen in a way that it is as simple as possible and as complex as necessary. However, validation of this model against invasive catheterization is necessary, which is work-in-progress. As the accuracy of catheter measurements are specified with 4 mmHg or 4% of the measured value, whichever is larger, the accuracy of the measured pressure gradient is approximately 8 mmHg, as it requires measurements up- and downstream the stenosis. We assume the inaccuracy introduced by the model choices to be below this inaccuracy. No information on the patient-specific flow fields within the ascending aorta was available. Therefore, a plug velocity profile was used as inlet boundary condition at the LVOT. In order to investigate, information on the patient-specific inlet profile that is usually derived from 4D velocity-encoded MRI information is required.

The proposed novel model requires information on two parameters: the patient-specific volume flow rate as well as AVA. Both parameters can be assessed using various non-invasive methods. The uncertainties of these parameters in clinical settings will have to be investigated. For the present study, earlier segmented geometries were used as ground truth. The same values for AVA and volume flow rate were used for simulation of the pressure gradient as well as the adjustment of the model. Therefore, the accuracy of those segmentations was not relevant for this study. However, accurate segmentation of the AV as well as calculation of the patient-specific, peak-systolic volume flow rate is essential for the clinical translation of the proposed method.

The current model was not validated using experiments with phantoms or by a comparison with clinically catheter-measured pressure gradients. The future clinical validation study may find inaccuracies of the model requiring further model optimization.

## Conclusion

A novel approach for accurate assessment of TPG across the AV based on volume flow rate and AVA was proposed. Both required parameters can be assessed by non-invasive methods, which are applied routinely. Before clinical translation of the proposed model, further studies with a larger cohort, including severe stenosis cases, and complex valve shapes, including bicuspid AVs, are required for model refinement. Furthermore, validation against catheter-based measurements will allow quantification against a routine invasive measurement.

## References

[1] Abraham F, Behr M, Henkenschloss M (2005). Shape optimization in steady blood flow: a numerical study of non-Newtonian effects. Comput Methods Biomech Biomed Eng.

[2] Batchelor GK (1967) An introduction to fluid dynamics. Cambridge University Press. isbn:978-0-521-66396-0

[3] Baumgartner H, Hung J, Bermejo J et al Echocardiographic assessment of valve stenosis: EAE/ASE recommendations for clinical practice. Eur J Echocardiogr 10(1):1–25 10.1093/ejechocard/jen303 19065003

[4] Bitar J, Douthat L, Alam M, Rosman HS, Lebeis M, Goldstein S, Khaja F (1990). Practical value of echo Doppler evaluation of aortic and mitral stenosis: a comparative study with cardiac catheterization. Henry Ford Hosp Med J.

[5] Chanson H (2004) Hydraulics of open channel flow: an introduction, 2nd edn. Butterworth–Heinemann ISBN 978-0-7506-5978-9, 650 pp

[6] Danielsen R, Nordrehaug JE, Stangeland L, Vik-Mo H (1988). Limitations in assessing the severity of aortic stenosis by Doppler gradients. Br Heart J.

[7] Donati F, Myerson S, Bissell MM (2017). Beyond Bernoulli: improving the accuracy and precision of noninvasive estimation of peak pressure drops. Circ Cardiovasc Imaging.

[8] Dyverfeldt P, Hope MD, Tseng EE, Saloner D (2013). Magnetic resonance measurement of turbulent kinetic energy for the estimation of irreversible pressure loss in aortic stenosis. J Am Coll Cardiol Img.

[9] Ecabert O, Peters J, Walker MJ, Ivanc T, Lorenz C, von Berg J, Lessick J, Vembar M, Weese J (2011). Segmentation of the heart and great vessels in CT images using a model-based adaptation framework. Med Image Anal.

[10] Garcia D, Kadem L (2006). Aortic valve area. J Heart Valve Dis.

[11] Goli VD, Teague SM, Prasad R, Harvey J, Voyles WF, Olson EG, Schechter E, Thadani U (1988). Noninvasive evaluation of aortic stenosis severity utilizing Doppler ultrasound and electrical bioimpedance. J Am Coll Cardiol.

[12] Goubergrits L, Riesenkampff E, Yevtushenko P, Schaller J, Kertzscher U, Hennemuth A, Berger F, Schubert S, Kuehne T (2015). Magnetic resonance imaging based computational fluid dynamics for diagnosis and treatment prediction: clinical validation study in patients with coarctation of aorta. J Magn Reson Imaging.

[13] Harrison MR, Gurley JC, Smith MD, Grayburn PA, DeMaria AN (1988). A practical application of Doppler echocardiography for the assessment of severity of aortic stenosis. Am Heart J.

[14] Hegrenaes L, Hatle L (1985). Aortic stenosis in adults: non-invasive estimation of pressure differences by continuous wave Doppler echocardiography. Br Heart Jf.

[15] Heys JJ, Holyoak N, Calleja AM, Belohlavek M, Chaliki HP (2010). Revisiting the simplified bernoulli equation. Open Biomed Eng J.

[16] Hoeijmakers (2019). Estimation of valvular resistance of segmented aortic valves using computational fluid dynamics. J Biomech.

[17] Iung B, Baron G, Butchart EG, Delahaye F, Gohlke-Bärwolf C, Levang OW, Tornos P, Vanoverschelde JL, Vermeer F, Boersma E, Ravaud P, Vahanian A (2003). A prospective survey of patients with valvular heart disease in Europe: the euro heart survey on valvular heart disease. Eur Heart J.

[18] Karimi S, Dabagh M, Vasava P, Dadvar M, Dabir B, Jalali P (2004). Effect of rheological models on the hemodynamics within human aorta: CFD study on CT image based geometry. J Non-Newton Fluid Mech.

[19] Lima CO, Sahn DJ, Valdes-Cruz LM, Goldberg SJ, Barron JV, Allen HD, Grenadier E (1983). Noninvasive prediction of transvalvular pressure gradient in patients with pulmonary stenosis by quantitative two-dimensional echocardiographic Doppler studies. Circulation.

[20] Miyazaki S, Itatani K, Furusawa T, Nishino T, Sugiyama M, Takehara Y, Yasukochi S (2017). Validation of numerical simulation methods in aortic arch using 4D flow MRI. Heart Vessel.

[21] Nguyen T-Q, Hansen KL, Bechsgaard T, Lönn L, Jensen JA, Nielsen MB (2019). Non-invasive assessment of intravascular pressure gradients: a review of current and proposed novel methods. Diagnostics.

[22] Nishimura RA, Otto CM, Bonow RO, Carabello BA, Erwin JP, Guyton RA, O'Gara PT, Ruiz CE, Skubas NJ, Sorajja P, Sundt TM, Thomas JD, ACC/AHA Task Force Members (2014). 2014 AHA/ACC guideline for the management of patients with valvular heart disease: executive summary a report of the American College of Cardiology/American Heart Association Task Force on Practice Guidelines. Circulation.

[23] Nkomo VT, Gardin JM, Skelton TN, Gottdiener JS, Scott CG, Enriquez-Sarano M (2006). Burden of valvular heart diseases: a population-based study. Lancet.

[24] Nobili M, Morbiducci U, Ponzini R, Del Gaudio C, Balducci A, Grigioni M, Montevecchi FM, Redaelli A (2008). Numerical simulation of a bileaflet prosthetic heart valve using a fluid-structure interaction approach. J Biomech.

[25] Nordmeyer S, Hellmeier F, Yevtushenko P, Kelm M, Lee CB, Lehmann D, Kropf S, Berger F, Falk V, Knosalla C, Kuehne T, Goubergrits L (2019). Abnormal aortic flow profiles persist after aortic valve replacement in the majority of patients with aortic valve disease: how model-based personalized therapy planning could improve results. A pilot study approach. Eur J Cardiothorac Surg.

[26] Oshinski JN, Parks WJ, Markou CP, Bergman HL, Larson BE, Ku DN, Mukundan S, Pettigrew RI (1996). Improved measurement of pressure gradients in aortic coarctation by magnetic resonance imaging. J Am Coll Cardiol.

[27] Otto CM (2006). Valvular aortic stenosis: disease severity and timing of intervention. J Am Coll Cardiol.

[28] Rijsterborgh H, Roelandt J (1987). Doppler assessment of aortic stenosis: Bernoulli revisited. Ultrasound Med Biol.

[29] Rosenhek R, Zilberszac R, Schemper M, Czerny M, Mundigler G, Graf S, Bergler-Klein J, Grimm M, Gabriel H, Maurer G (2010). Natural history of very severe aortic stenosis. Circulation.

[30] Schwinger ME (1991). Doppler echocardiography versus cardiac catheterization in the evaluation of valvular heart disease: do we have a gold standard?. Clin Cardiol.

[31] Soler-Soler J, Galve E (2000). Worldwide perspective of valve disease. Heart.

[32] Weese J, Lungu A, Peters J, Weber FM, Waechter-Stehle I, Hose DR (2017). CFD- and Bernoulli-based pressure drop estimates: a comparison using patient anatomies from heart and aortic valve segmentation of CT images. Med Phys.

[33] Xu X, Liu TY, Li C, Zhu L, Li SX (2019). A numerical analysis of pressure pulsation characteristics induced by unsteady blood flow in a bileaflet mechanical heart valve. Processes.

[34] Yoganathan AP, Cape EG, Sung HW, Williams FP, Jimoh A (1988). Review of hydrodynamic principles for the cardiologist: applications to the study of blood flow and jets by imaging techniques. J Am Coll Cardiol.

